# Case of Facial Paralysis

**Published:** 1873-09

**Authors:** Gouverneur M. Smith


*Case of Facial Paralysis.* By GOUVERNEUR M. SMITH, M.D. (Read before the New York Academy of Medicine, June 5, 1873.)

The brief recital of a case of Bell's paralysis, which has recently come under my care, may prove of interest, as the method of treatment adopted tends to show the correctness of views originally presented to this Academy a short time since by one of its distinguished fellows.

On the 5th of April last, a patient came under my care, suffering with paralysis of the left side of the face. The patient was a gentleman of culture and means, about sixty years of age. Residing for a large part of the year at his country-seat, on the Hudson, and spending much of the time in the open air, he was ordinarily in the enjoyment of excellent health, and manifested his robust condition by a commanding appearance. The occurrence of such local palsy was the occasion of no little solicitude in the mind of the patient, lest it be precursory of a hemiplegic seizure.

In studying the etiology of the malady, it seemed probable that the disorder had been excited by cold, to which the patient had been exposed while riding in the Central Park on the day previous to the one upon which the paralysis was fairly developed. There was no evidence of centric disturbance; peripheral lesion was not marked by any decided local point of irritation.

In speaking of peripheral facial hemiplegia, Aitken remarks: "Although it is not a dangerous form of paralysis, it is one from which recovery is very slow, and in which prognosis, as to complete recovery of symmetry of the face, is uncertain;" and also says, "from four to ten months is the ordinary duration of the affection; but there are instances in which the paralysis yields in twenty-four, fifteen, or even twelve hours, but such cases are exceptional" (Trousseau).

After regulating the bowels of my patient, he was placed under the use of iodide of potassium, and on four occasions electrization by a specialist was applied to the affected side. No counter-irritation behind the ear was resorted to, owing to the absence of apparent local lesion. On the 21st of April the patient had shown little or no improvement. I remembered that Dr. William Detmold had read a paper before this Academy (March 20, 1873,)

entitled "Facial Paralysis treated by a New Method." Not having been present at the reading of the paper, I called upon Dr. Detmold, and he briefly gave me the views he had here expressed, and as since published in the *New York Medical Journal*, May, 1873.

In the case which he has reported he says: "I determined to try what mechanical means would do. I bent a wire into a hook, which I put into the drooping corner of the mouth, and drawing it up, bent the wire over and behind the ear. I recommended the patient to keep it on over night, trusting that, by entirely relaxing the paralyzed muscles, and supporting the dragging weight, I might somewhat relieve the defect." Prompt amelioration followed this method of treatment. Dr. Detmold further says: "It then occurred to me that I might make this instrument still more effective, if I could combine with it a permanent and continuous galvanic current through the paralyzed parts, by having it made of two different metals, thus forming as it were a single cell of a galvanic battery." An instrument fulfilling such purpose was made by Mr. Chester under Dr. Detmold's direction, and the patient at the time of the report was steadily improving. The case had been a chronic one, of sixteen years' duration, and had not before been relieved. In his conclusion Dr. Detmold remarks: "I am unable to say what share in the benefit, or whether any, is due to the galvanic current, to which, on the whole, I do not attach as much importance as to the mechanical support."

Resolving to test the applicability of this method of treatment to the acute case under my care, I procured from Mr. Stohmann a German-silver wire mouth-piece, used by the dentists in holding the mouth open during dental operations. The dentists employ two, one on each side. One of these I bent in such a manner that it would not keep the mouth open, but simply act as a hook comfortably catching the corner of the mouth, and to the outer end fastened a piece of copper wire, which, passing across the cheek, was turned around the ear. The wire passing over the ear being covered with a soft material, was not a source of irritation. As the cheek was quite pendulous, I ordered a bandage to be passed around the head under the jaw, to give additional support.

The relief which followed was significant, for, after using this appliance for two nights, decided amelioration was manifest. Wishing the patient to avail himself of any advantage that might be derived from the galvanic current, I went with him to Mr. Charles T. Chester's, 104 Centre Street, and, giving a wire model as to size, Mr. Chester had prepared this neat instrument, which is a fac simile in principle of the one made under Dr. Detmold's direction. The smooth and easily fitting hook or mouth-piece is made of platinum; the wire running across the cheek and turning behind the ear is of silver, and to this is adapted a zinc plate, which is covered with velvet, with the view of readily retaining the moisture of either saline, acidulated, or pure water.

The patient on procuring this instrument substituted it for the one I had extemporized, using it at night; convalesence was rapid. Recovery from the facial paralysis was complete in about a month from the time of its incipiency. There has been no recurrence of the difficulty; the symmetry of the face is normal.

Several questions naturally arise in this connection. In the first place, was this case one of those occasionally met with, in which recovery takes place without material artificial assistance? and, in the second place, if recovery is due to treatment, how far was it attributable to the mechanical means, and how far to galvanism? In response I would say that, there was scarcely any perceptible improvement in the patient until the "mechanical means" were resorted to; convalescence seemed to date from the night they were employed.

Whether or not the second instrument was a more potent remedial factor, by its galvanic properties, it is difficult to say. The patient was not conscious of any galvanic influence, though there is no question of the passage of a current through the affected side, by means of this appliance, but, as stated in Dr. Detmold's paper, from the periphery to the centre. In regard to the action of this instrument, Mr. Chester has written to me as follows: "I have tested the little galvanic battery, made to apply to the face of Mr. ——, in a general way. The covering of the zinc plate, being moistened with water, made a good conductor by the addition of a slight trace of acid, and the plate then applied to (behind) the ear, while the platinum end was inserted in the mouth, I find that it generates a steady current capable of deflecting a galvanometer or sending a telegraph message easily through seventy-five miles of the ordinary telegraph wire."

This case, so far as I am aware, is the first acute one treated by the method suggested by the distinguished fellow to whom allusion has been made.—*New York Medical Journal.*

